# A socio-psychological model of laser levelling impacts assessment

**DOI:** 10.1186/s40504-020-0097-2

**Published:** 2020-02-17

**Authors:** Somayeh Tohidyan Far, Kurosh Rezaei-Moghaddam

**Affiliations:** Department of Agricultural Extension and Education, School of Agriculture, Shiraz University, Shiraz, Iran

**Keywords:** Laser land levelling, Environmental impact assessment, Structural equation modeling, Iran

## Abstract

Application of technologies has an important role in agricultural development. Identifying and assessing the impacts of agricultural technologies is necessary. This study aimed at assessing the impacts of laser levelling economically, socially, environmentally, and technically in the viewpoint of the agricultural experts and identifying factors determining their perception of the impacts. The study samples (151 experts) were selected using multi-stage random sampling in Fars Province, Iran. The results revealed that experts considered uniform distribution of water, using conservation tillage, facilitating agricultural activities, decreased water consumption and decrease of water wasting as the most important technical impacts of laser levelling technology. The most environmentally important impacts were the decrease of soil erosion and retention of crop residues. Experts stated the most significant social impacts as improvement in villages living conditions and sense of belonging to rural areas. Besides, an increase of income and reduction of inputs costs were among the economic impacts of laser levelling technology. According to the results, attitude towards water and soil resources conservation and environmental beliefs had the highest direct effect on individual perception toward impacts. Practical recommendations have been presented based on the results of the study.

## Introduction

Previous studies revealed that agricultural inputs such as soil, water, chemical fertilizers, seeds, agricultural machines and human resources are not used in uneven lands in an optimized way (Das et al. 2018; Tajer et al. 2010; Sattar et al. 2003; Jat et al. 2006). Agricultural technologies have several significant impacts on the economy and society locally, regionally and nationally from different aspects (Yang 2005). It is necessary to assess the impacts for conscious decision-making in order to revise them appropriately (Koszalka and Grabowski 2003). Assessing the impacts of agricultural technologies is necessary for maximizing benefits and minimizing negative consequences. Planning for equilibrium development requires economic, social, environmental and technical impacts to be taken into consideration (Pasakarnis and Maliene 2010).

Social, economic and biophysical impacts are inherently and inextricably interconnected. Social impact assessment develops an understanding of the impact pathways, when a change in one domain triggers impacts across other domains, as well as the iterative or a flow-on consequences within each domain (Vanclay 2003). Environmental Impact Assessment means that the effects of development actions can be identified and evaluated in advance (Glasson et al. 2005). Environmental impacts refer to variations made as a result of different activities in physical environment (climate, land and soil), ecology (quality and quantity of surface water, air, sound and soil), biology (animal and plants species, sensitive environmental areas, natural habitats, diseases vectors), and social-economy (population, education, specialty, income, facilities, employment, sanitation, health, views and landscape) (Memari and Soleimani 2006).

## Main text

Previous studies have been reported assessing the impacts of the laser land levelling technology. Different studies have confirmed that laser levelling technology will decrease farming costs in different cultivation and harvest stages (Abdullaev et al. 2007; Gulati et al. 2017). Laser land levelling causes the reduction of pesticides consumption, improves the use of nutritious materials and reduces consumption of chemical fertilizers (Abdullaev et al. 2007; Jat et al. 2006; Gonzalez et al. 2009). Decreasing the amount of water consumption, uniform distribution of water, reducing irrigation frequency and time and water wasting are among the most important impacts (Abdullaev et al. 2007; Gonzalez et al. 2009; Das et al. 2018; Jehangir et al. 2007; Shahani et al. 2016; Ashraf et al. 2017). Reducing the use of seeds, uniformity of germination and crop growth and increasing yield have been mentioned in some studies (Abdullaev et al. 2007; Jehangir et al. 2007; Jat et al. 2006). Jat et al. (2006) noted that the amount of fuel consumed by pump engine for pumping water and agricultural machinery would be reduced by this technology. Some studies showed that after laser levelling farmers consider their Crete bigger than before it (Rickman 2002; Jat et al. 2006). Also, land levelling led to an increase in the cultivable area (farm useful area) and under-cultivated area based on accessible water supply. Abdullaev et al. (2007) and Jat et al. (2006) indicated that farmers’ income will be increased by levelling lands. Other impacts of land levelling are reducing family workforce and the number of laborers needed for different farming operations (Abdullaev et al. 2007; Akhtar 2006).

Juarez-Najera et al. (2009) presented a social-psychological model for determining sustainable behaviors. This model focused on values and moral norms, rather than rational choice and self-interest. They considered environmentally friendly behaviors, as an evolving concept from environmental psychology and sustainability perspectives. The focus of environmental psychology is on the relationship between human and the broader environment. According to this study, ascription of responsibility and awareness of consequence will inform us of people’s desire for solving environmental problems. They studied different models and found that self-enhancement values, self- transcendence values, conservation values, and openness to change values would affect individual awareness of environmental consequences. Gonzalez Lopez and Cuervo-Arango (2008) examined the relationship between psychological structures and environmental behavior. The results indicated that biospheric values affected the awareness of environmental consequences positively and directly. On the other hand, environmental beliefs influenced the awareness of consequences negatively and directly.

The results of Hansla et al. (2008) stated that the awareness of the consequences and environmental concerns was related to benevolence values, power, and universalism. They showed that each environmental consequence was related to a kind of value significantly (awareness of consequences of environmental problems for themselves with power, awareness of consequences for biosphere with universalism and awareness of consequences for others with benevolence). Also, social, egotistic, biospheric environmental concerns were related to their corresponding awareness of consequences.

According to Schwartz’s theory, the awareness of the consequences was one of the main factors to determine environmental behaviors. Stern et al. (1999) consider the difference between egoistic, social-altruistic and biosphere awareness of consequences. Based on these consequences values will be directed. “The awareness of consequences must induce an ascribed responsibility to perform the behavior that in turn activates a personal norm or moral obligation to perform the behavior” (Garling et al. 2001).

Van Liere and Dunlap (1987) revealed a significant relationship between taking responsibility and environmental behavior while there was a weak correlation between the awareness of consequences and environmental behaviors. In addition, there was a mutual relationship between taking responsibility, the awareness of consequences, and environmental behaviors. According to Schwartz theory, behavior will be formed based on internal relationships among social norms, personal norms, the awareness of environmental consequences and individual responsibility-taking (Qashu 2007).

According to Stern et al. (1999) sustainable behavior is due to personal norms activation by individual beliefs and values. Norm-belief-value originates from Altruism behavior theory. Responsibility has great importance in theory and will directly determine behavior. Personal norms are set by individual awareness of positive consequences resulting from activities and responsibilities. These two variables affect behavior directly. Based on Schwartz’s theory, norms will be activated when an individual has two kinds of beliefs, including the awareness of behavioral consequences and taking responsibility towards consequences provision and prevention.

Ibtissem (2010) defined conservative behaviors as an aspect of sustainable behavior by norm-belief-value theory. He distinguished environmental values from social values. Social values represent the individual’s relationship with oneself, inside groups and others, while environmental values reflect the human being’s relationship to the natural environment. Moreover, he made a difference between anthropocentric values and eco-centric values to evaluate environmental values. The results of the study stated the positive and direct impact of anthropocentric values on the individual awareness of consequences. Ryan and Spash (2010) used environmental concerns and the awareness of consequences. The results showed that biospheric concerns about environmental problems had a negative relationship with the individual belief, which indicated that the environment would not be harmed due to human activities. Also, egoistic and social concerns had a positive relationship with the belief of negative consequences of human activities for the environment. Increase of knowledge leads to improved attitudes and behavioral intention and it can be a mediator between attitudes and behavior. It changes attitudes and, finally, behavior (Kalantari and Abdollahzadeh 2008).

Since 2004, laser land levelling has been initiated for increasing the productivity of water and soil resources, conserving soil, providing a balance in underground water resources, increasing farming products, decreasing the consumption of different kinds of chemical fertilizers and agricultural pesticides, performing water and soil infrastructure rapidly and preserving agricultural products health standards (Fars Province Laser land levelling Strategic Committee 2007). This study aimed at assessing the impacts of laser levelling economically, socially, environmentally, and technically in the viewpoint of the agricultural experts and identifying factors determining their perception of the impacts.

## Research method

The survey was used in this study among experts working in Fars Province Agriculture Jihad Organization. According to Kerjcie and Morgan (1970) using multi-stage random sampling, 151 experts were selected. The questionnaire was used to collect data. Indicators were determined through three steps. In the first step, based on documents, information related to the impacts of laser land levelling technology was gathered. In the second step, for confirming the impacts, pioneer farmers (6 farmers) domiciled at Zarghan and Marvdasht regions were interviewed. The farmers were deeply interviewed with open ended questions. In the last step some experts of Mechanization Departments and Water and Soil Department of Agriculture Jihad Organization in Fars Province as the technology executives, were interviewed. Eventually, the findings obtained from these steps were conceptualized and more frequent indicators were considered as the impacts of the technology. In order to measure the impacts of laser levelling technology, 83 questions were designed in the fields of technically, socially, economically and environmentally.

### Variables measurement

#### Environmental concern

This variable was estimated using items related to environmental concern toward valuing objects that are representative of egoistic, altruistic and biospheric value orientations (Schultz 2001; Schultz et al. 2004).

#### Taking responsibility for water and soil conservation

This variable measures the responsibility and obligation of experts for protecting soil and water resources, improving soil and water resources, informing farmers about the dangers of soil and water resource degradation, etc.

#### Attitude toward water and soil conservation

This was measured by items related to the experts’ opinions toward protecting soil and water resources, flood control, the importance of soil and water resources, water quality and quantity, etc.

#### Environmental beliefs

This was estimated using five items (consisting of 15 Likert-scale items) from Dunlap’s longer scale (Dunlap et al. 1992), such as: When humans interfere with nature, it often produces disastrous consequences, we are approaching the limit of the number of people the Earth can support; Humans have the right to modify the natural environment to suit their needs.

#### Spirituality

It is related to values and beliefs “that gives one’s life meaning and direction” (Kolodinsky 2010). This was measured using items related to looking for comfort and relaxation in nature, spending a day in nature as a spiritual experience, etc.

#### Social responsibility

“The obligations of expert pursue those policies, to make those decisions, or to follow those lines of action which are desirable in terms of the objectives and values of our society” (Carroll 1999). This variable was estimated using items related to providing a better environment for future generations, the world needs responsible people, etc.

#### Impacts of technology

To measure this variable, 83 indicators were asked in the field of environmental, social, technical, and economic impacts.

#### Knowledge of laser levelling technology

This was measured by items related to experts’ technical knowledge of laser levelling and activities required to manage the land before and after the laser levelling.

The validity of questionnaire was tested by the opinions of professors at Shiraz university and experts in Agriculture Jihad Organization in Fars Province. A pilot-test was conducted using a sample of 30 experts outside the study area. The questionnaire was improved based on the pilot study. Cronbach’s alpha was used for measurement (Table 1). Data was analyzed by SPSS and LISREL soft wares, versions 16 and 8.54 respectively.

**Table 1:** Cronbach’s alpha coefficients for research variables

Variables	Cronbach’s alpha coefficients
Environmental concerns (Social/Altruistic)	0.69
Environmental concerns (Biospheric)	0.68
Taking responsibility for water and soil conservation	0.81
Attitude towards water and soil resources conservation	0.75
Environmental beliefs	0.78
Spirituality	0.70
Social responsibility	0.85
Impacts of technology	0.91
Knowledge of laser levelling technology	0.76

### Scope of the study

**Fig. 1:**
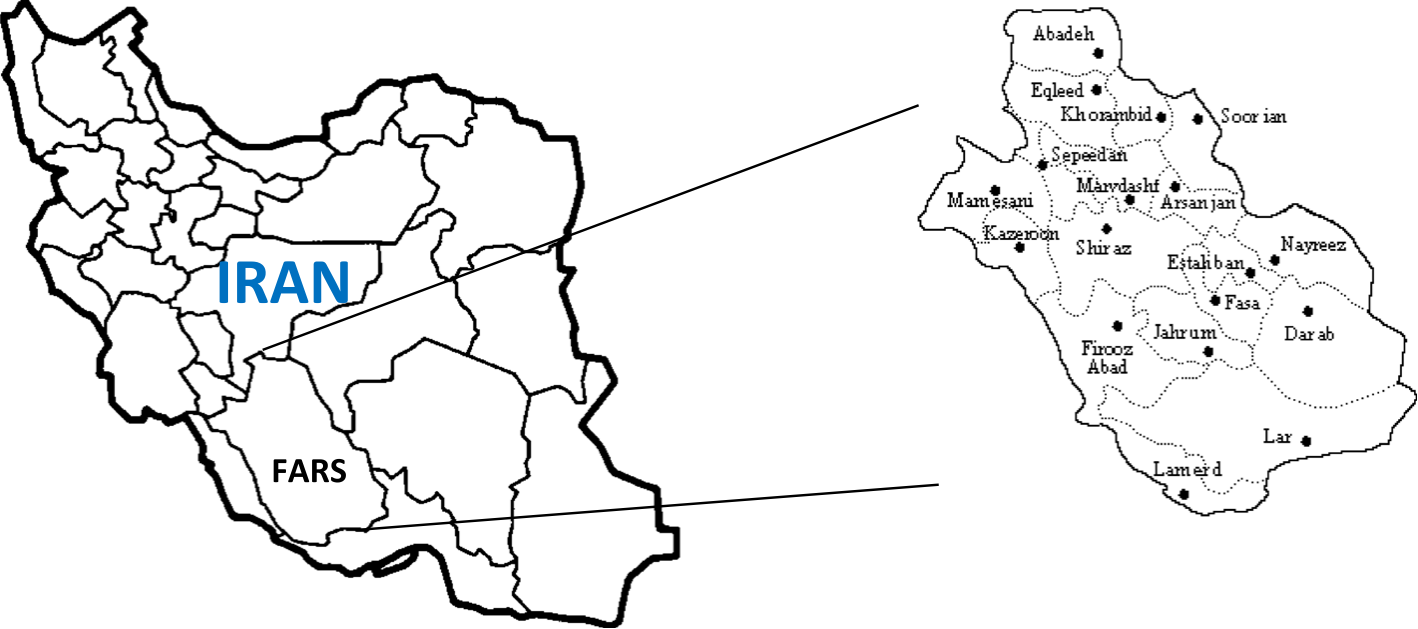
A general map of Iran illustrating the location of the study area

This study was carried out in Fars Province, Iran. The majority of annual water production of Iran belongs to Fars Province, which includes 11.83% of the water level of the country. About 9.7% of total agricultural products is in this level. Climate variation, agricultural farming lands expansion, the existence of long records and agricultural science centers provide acceptable status in the agriculture of this province and a proper capability for expanding agricultural technologies qualitatively and quantitatively. Based on the high level of water products in this province and water crisis, Fars Province is one of the pioneers in the introduction and application of laser land levelling technology in Iran. Application of this technology was started in 6 ha in this province in 2004 and reached to 225,000 ha by the year 2016. The geographical status of this province is shown in Fig. 1.

## Results and discussion

### Technical impacts of laser Levelling

Technical impacts of laser levelling can be examined in five categories including water consumption, yield management, mechanization, good farming, and farm area expansion. Table 2 demonstrates frequency and ranking mean of each impact of this technology. Due to ranking mean of technical impacts of laser levelling, experts consider uniform distribution of water, the ranking mean of which is equal to 2.73, as the most important technical impact. As seen in the table, 76.8% of experts assessed that the impact of laser levelling on uniform distribution of water was high and only 4% believed that laser levelling had low impact on uniform distribution of water. Using conservation tillage with ranking mean 2.57 has the second rank and 54.3% stated the impact of laser levelling on using conservation tillage was high, while 28.5% assessed it average. Only 2.6% of experts claimed that laser levelling had low impact on using conservation tillage. Also, 64.2% of experts firmly believed that laser levelling resulted in uniform growth of crops and just 1.3% stated laser levelling had no impact on uniform growth of crops. Rank mean of uniform growth of the crop was 2.56. The results showed that ranking means of facilitating agricultural activities, decrease of water consumption, decrease of water wasting, uniform germination of crops are 2.54, 2.53, 2.52, and 2.50, respectively. Decrease of water consumption and water-wasting are significant impacts of laser levelling in Iran, which is facing water shortage crisis.

**Table 2:** Technical impacts of laser land levelling technology

Indicators	Priority	Rank mean	Positive impact						No impact	
			Low		Average		High			
			Frequency	Percent	Frequency	Percent	Frequency	Percent	Frequency	Percent
Water consumption										
Water consumption	5	2.53	11	7.3	47	31.1	91	60.3		
Irrigation time	11	2.40	11	7.3	57	37.7	78	51.7	3	2.0
Uniform distribution of water	1	2.73	6	4.0	27	17.9	116	76.8		
Irrigation method	14	2.36	16	10.6	47	31.1	81	53.6	5	3.3
Frequency of irrigation	20	2.26	18	11.9	50	3.1	73	48.3	8	5.3
Deep percolation of water	15	2.35	17	11.3	52	34.4	76	50.3	3	2.0
Water waste	6	2.52	13	8.6	33	21.9	99	65.6	4	2.6
Moisture-holding capacity	24	2.14	16	10.6	59	39.1	62	41.1	12	7.9
Underground waters level	31	1.91	27	17.9	51	33.8	52	34.4	19	12.6
Yield management										
Uniform germination of crop	7	2.50	12	7.9	44	29.1	92	60.9	2	1.3
Uniform growth of crop	3	2.56	8	5.3	43	28.5	97	64.2	2	1.3
Crop yield	16	2.34	13	8.6	60	39.7	73	48.3	4	2.6
Crop shedding	19	2.28	17	11.3	45	29.8	78	51.7	9	6.0
Yielding period length	39	1.59	24	15.9	45	29.8	41	27.2	39	25.8
Density of crop	25	2.08	20	13.2	58	38.4	58	38.4	13	8.6
Turnaround time between harvesting one crop and planting another	36	1.64	21	13.9	50	33.1	41	27.2	36	23.8
Planting date	37	1.63	21	13.9	47	31.1	43	28.5	38	25.2
Harvest date	34	1.67	24	15.9	44	29.1	46	30.5	35	23.2
Increasing positive competition for more production	31	1.91	26	17.2	62	41.1	45	29.8	16	10.6
Mechanization										
Improving machineries traverse (Improving field trafficability)	13	2.37	10	6.6	46	30.5	84	55.6	8	5.3
Machineries speed (time saving)	8	2.48	12	7.9	43	28.5	90	59.6	3	2.0
Traffic of tractor in the field	21	2.25	23	15.2	41	27.2	76	50.3	8	5.3
Using zero tillage planting	14	2.36	19	12.6	41	27.2	84	55.6	5	3.3
Using Conservation tillage	2	2.57	19	12.6	43	28.5	82	54.3	4	2.6
Using chopper-combine	19	2.28	19	12.6	51	33.8	73	48.3	6	4.0
Combinate use	18	2.32	23	15.2	42	27.8	80	53.0	4	2.6
Using modern solutions sprayer	22	2.19	17	11.3	50	33.1	70	46.4	12	7.9
Time taken for land preparation	23	2.16	22	14.6	44	29.1	70	46.4	12	7.9
Time taken for planting	24	2.14	17	11.3	52	34.4	67	44.4	14	9.3
Time taken for harvesting	11	2.40	12	7.9	51	33.8	71	47.0	15	9.9
Good farming										
Facilitating agricultural activities	4	2.54	12	7.9	45	29.8	93	61.6		
Farm infrastructures	29	1.97	25	16.6	50	33.1	57	37.7	18	11.9
Cropping pattern	35	1.66	27	17.9	44	29.1	44	29.1	33	21.9
Crop rotation	38	1.60	30	19.9	40	26.5	43	28.5	36	23.8
Land fallow	40	1.52	24	15.9	40	26.5	41	27.2	43	28.5
Crop diversity	38	1.60	27	17.9	41	27.2	44	29.1	38	25.2
Tillage operation	17	2.33	16	10.6	58	38.4	72	47.7	3	2.0
Land consolidation	10	2.42	12	7.9	44	29.1	88	58.3	6	4.0
Pesticide consumption	36	1.64	39	25.8	40	26.5	43	28.5	29	19.2
Number of spraying pesticides	33	1.70	33	21.9	47	31.1	43	28.5	27	17.9
Fertilizer consumption	27	1.98	26	17.2	53	35.1	55	36.4	16	10.6
Seed consumption	26	2.07	30	19.9	54	35.8	57	37.7	8	5.3
Fossil fuel consumption	32	1.89	37	24.5	57	37.7	45	29.8	12	7.9
Electricity use	30	1.92	32	21.2	50	33.1	53	35.1	16	10.6
Farm area expansion										
Crete’s sizes	9	2.44	11	7.3	48	31.8	85	56.3	4	2.6
Expansion of total area planting	12	2.39	11	7.3	59	39.1	76	50.3	3	2.0
Cultivable area (Farm useful area)	11	2.40	17	11.3	48	31.8	81	53.6	2	1.3
Cultivation in barren lands	19	2.28	19	12.6	51	33.8	73	48.3	6	4.0

Table 2 showed 28.5% of experts assessed that this technology had no impact on land fallow and 26.5% believed that this technology caused farmers to leave their lands fallow. This technical impact has the raking mean of 1.52 so that experts consider it as the least laser levelling impact. Also, it is seen that 25.8% of experts believed that laser levelling will not change yielding period length and 29.8% explained it influences yielding period length at average level. The mean of this impact was 1.59. Other technical impacts have been shown in Table 2.

### Environmental impacts of laser Levelling

The environmental impacts of laser levelling are classified into being vulnerable against disasters, soil production capacity, crop residues management, pollution, and biodiversity. The experts assessed decrease of soil erosion and retention of crop residues, with ranking mean equal to 2.26, as the most important environmental impacts of laser levelling. The distribution results of soil erosion and retention of crop residues revealed that 50.3 and 45% of experts assessed the high impact of laser levelling on the decrease of soil erosion and retention of crop residues. While only 6.6 and 4.6% believed that laser levelling had no effect on the decrease of soil erosion and retention of crop residues. Reduction in the number of pests was another impact stated by experts so that it decreased pesticide use significantly. This item with ranking mean of 2.22 took the second rank. 48.3% of experts believed that laser levelling could greatly increase soil fertility and only 8.6% believed that this technology had no impact on increase of soil fertility. Ranking mean of this indicator was 2.18 and it took the third rank as well as decrease of weeds density with ranking mean of 2.18. Further, 45% of experts stated that laser levelling decreased weeds density in large amount. According to Table 3, village landscape attractiveness and coping with drought were considered as important impacts with ranking mean of 2.13 and 2.12, respectively. Authorities and experts have always considered laser levelling as one of the strategies for coping with drought.

**Table 3:** Environmental impacts of laser land levelling technology

Indicators	Priority	Rank mean	Positive impact						No impact	
			Low		Average		High			
			Frequency	Percent	Frequency	Percent	Frequency	Percent	Frequency	Percent
Vulnerable against disasters										
Facilitated pest control	2	2.22	22	14.6	51	33.8	70	46.4	7	4.6
Weeds density	3	2.18	19	12.6	52	34.4	68	45.0	11	7.3
Coping with drought	5	2.12	22	14.6	46	30.5	67	44.4	13	8.6
Soil production capacity										
Soil fertility	3	2.18	20	13.2	44	29.1	73	48.3	13	8.6
Soil erosion	1	2.26	14	9.3	50	33.1	76	50.3	10	6.6
Soil compaction	7	1.76	29	19.2	43	28.5	50	33.1	28	18.5
Soil salinity	8	1.74	23	15.2	45	29.8	49	32.5	32	21.2
Residues management										
Retention of crop residues	1	2.26	15	9.9	60	39.7	68	45.0	7	4.6
Mixing the soil with crop residues	6	2.04	21	13.9	53	35.1	59	39.1	16	10.6
Pollution										
Underground waters pollution	9	1.63	32	21.2	44	29.1	42	27.8	32	21.2
Fossil fuel pollution	10	1.61	37	24.5	37	24.5	45	29.8	30	19.9
Biodiversity										
Terricolous organisms diversity	11	1.56	32	21.2	40	26.5	40	26.5	36	23.8
Beneficial insect attraction	12	1.33	27	17.9	32	21.2	34	22.5	52	34.4
landscape attractiveness	4	2.13	22	14.6	56	37.1	62	41.1	10	6.6

### Social impacts of laser Levelling

Social impacts of this technology can be classified to job opportunities, sense of belonging to the village (place attachment), immigration, and welfare. As it is seen in Table 4, experts believed that improving farmers’ living conditions as the most important social impact, having a ranking mean of 1.96. As seen, 35.8% of experts assessed high impact of laser levelling on farmers’ living condition and 31.8% assessed it average. Only 7.3% believed that laser levelling had no effect on farmers’ living conditions. Based on the results, sense of belonging to village and quality of life satisfaction with ranking mean of 1.72 were placed after living condition improvement, and 31.1% and 27.8% of experts considered laser levelling as having high impact on increasing sense of belonging to village and quality of life satisfaction, while 28.5% and 29.8% stated an average impact.

**Table 4:** Social impacts of laser land levelling technology

Indicators	Priority	Rank mean	Positive impact						No impact	
			Low		Average		High			
			Frequency	Percent	Frequency	Percent	Frequency	Percent	Frequency	Percent
Job opportunities										
Seasonal agricultural labor	7	1.46	30	19.9	37	24.5	39	25.8	45	29.8
Employment opportunities	8	1.45	27	17.9	38	25.2	39	25.8	46	30.5
Number of family workforce	10	1.34	31	20.5	36	23.8	32	21.2	49	32.5
Sense of belonging to village (place attachment)										
Sense of belonging to village	2	1.72	30	19.9	43	28.5	47	31.1	29	19.2
Interest in living in villages in the future	3	1.71	37	24.5	47	31.1	42	27.8	24	15.9
Interest in spending most time in village	5	1.65	34	22.5	40	26.5	44	29.1	31	20.5
Taking responsibility for improving villages status	5	1.65	42	27.8	39	25.8	42	27.8	26	17.2
Immigration										
Immigration from rural areas	6	1.50	32	21.2	40	26.5	38	25.2	40	26.5
Immigrants returns	9	1.35	33	21.9	37	24.5	32	21.2	48	31.8
Welfare										
Improving farmers’ living condition	1	1.96	37	24.5	48	31.8	54	35.8	11	7.3
Quality of life satisfaction	2	1.72	42	27.8	45	29.8	42	27.8	21	13.9
Enjoying life	3	1.71	34	22.5	45	29.8	44	29.1	26	17.2
Spending more time with their families	4	1.67	38	25.2	50	33.1	37	24.5	24	15.9
Reduce poverty and inequality	7	1.46	31	20.5	46	30.5	32	21.2	41	27.2

Interest in living in rural areas and enjoying life and entertainments had a ranking mean of 1.71. Experts believed that using this technology caused they spend more time with their families due to working less and facilitating agricultural activities. This social impact with ranking mean of 1.67 was taken into consideration. Other social impacts of the technology were presented in Table 4.

### Economic impacts of laser Levelling

Experts assessed the most important economic impact of the technology as increase of net income and reduction of tillage cost with ranking mean of 2.17, so that 39.7% and 41.7% of the sample considered high impact of the technology on increase of net income and reduction of tillage cost and 40.4% and 36.4% assessed it averagely (Table 5). In this regard, increasing under cultivated lands, reducing costs of cultivation and harvest, changing cropping pattern (most farmers who leveled their lands, started to plant summer crops so that they could earn more money compared to winter crops), lessening the workload and workdays, and finally earning off farm income can be mentioned. After these impacts, rise in the price of land, with ranking mean 2.10, was important. The results showed that 39.1% of experts assessed the impact of laser levelling on land price significantly and 37.7% stated it averagely. Reduction in inputs cost and working days, with a ranking mean of 2.0 was placed at the lowest level. Ranking mean of this impact is 2.00 and the majority assessed the impact of laser levelling on input costs and reduction of working days high and average.

**Table 5:** Economic impacts of laser land levelling technology

Indicators	Priority	Rank mean	Positive impact						No impact	
			Low		Average		High			
			Frequency	Percent	Frequency	Percent	Frequency	Percent	Frequency	Percent
Net income	1	2.17	27	17.9	61	40.4	60	39.7	3	2.0
Tillage cost	1	2.17	30	19.9	55	36.4	63	41.7	3	2.0
Land price	2	2.10	27	17.9	57	37.7	59	39.1	8	5.3
Labor cost	3	2.04	29	19.2	58	38.4	54	35.8	9	6.0
Investment rate for agriculture	4	2.01	26	17.2	67	44.4	48	31.8	10	6.6
Number of working days	5	2.00	38	25.2	59	39.1	49	32.5	5	3.3
Inputs cost	5	2.00	33	21.9	57	37.7	52	34.4	9	6.0

In the review of literature on environmental impacts, there are direct and indirect impacts classifications. “Direct impacts, which are caused by the action and occur at the same time and place”. Indirect impacts result from the direct impacts and are defined as the reasonably foreseeable effects caused by the technology “later in time or farther removed in distance” (WSDOT Environmental Procedures Manual 2011). Network approach identifies the directions of direct and indirect environmental impacts (Tolba et al. 1987). Network diagrams provide a means for displaying first, secondary, tertiary, and higher order impacts. “The first step in network diagram is to identify the first order changes in environmental components. The secondary changes in other environmental components that will result from the first order changes are then identified. This process is continued until the network diagram is completed” (Lohani et al. 1997).

**Fig. 2:**
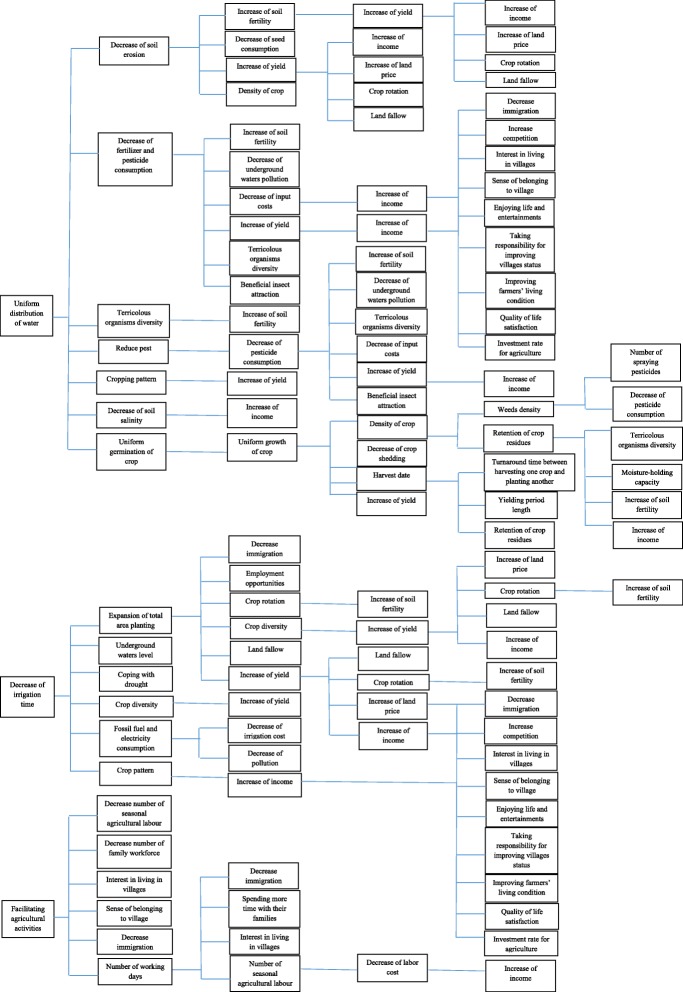
Causal chain of impacts of laser land levelling technology

In Fig. 2 the causal chain of some laser levelling impacts is presented. As it is seen, impacts are made in response to laser levelling immediately called “first impacts” or “direct impacts.” In addition, some impacts are brought about in response to the first impacts named “secondary impacts.” In this way, higher order impacts are made and also a change in one field may lead to changes in other fields. For example, some economic changes have resulted in social changes. As it is seen, uniform distribution of water is considered as a direct impact of laser levelling. Uniform distribution of water led to the secondary impacts of the technology such as decrease of erosion, reduction in pesticides and fertilizers consumption, terricolous organisms diversity, decrease in the number of pests, cropping pattern change, reduction of soil salinity and uniform germination of crop. Decrease of soil erosion causes an increase of fertility, decrease of seed consumption, increase of yield, and increase of crop density, thus the impacts of higher processes will be made. Increasing income is one of the economic impacts resulting in social impacts, including decrease of immigration, sense of belonging to the rural areas, enjoying life and entertainment, living condition improvement, quality of life satisfaction, etc.

### Relationships between variables

There was a significant correlation between individual perception of impacts and environmental concerns (social/altruistic), environmental concerns (biospheric), taking responsibility for water and soil conservation, attitude towards water and soil resources conservation, environmental beliefs, social responsibility and individual knowledge of laser levelling (Table 6). Moreover, there was a significant correlation between individual perception of the impacts and spirituality. A positive and significant correlation was seen between environmental beliefs and taking responsibility regarding water and soil resources conservation (*r* = 0.68), attitude towards water and soil resources conservation (*r* = 0.77), social responsibility (*r* = 0.56), and individual knowledge of laser levelling (*r* = 0.36). In addition, there is a significant relationship between social responsibility and taking responsibility regarding water and soil resources conservation (*r* = 0.49), attitude towards water and soil resources conservation (*r* = 0.46), and spirituality (*r* = 0.50).

**Table 6:** Correlation coefficients matrix between variables

Variable	Environmental concerns (Social/Altruistic)	Environmental concerns (Biospheric)	Taking responsibility for water and soil conservation	Attitude towards water and soil resources conservation	Environmental beliefs	Spirituality	Social responsibility	Individual perception of impacts	Knowledge of laser levelling
Environmental concerns (Social/Altruistic)	1								
Environmental concerns (Biospheric)	0.82^**^	1							
Taking responsibility for water and soil conservation	0.22^**^	0.20^*^	1						
Attitude towards water and soil resources conservation	0.03	0.03	0.61^**^	1					
Environmental beliefs	0.27^**^	0.26^**^	0.68^**^	0.77^**^	1				
Spirituality	0.37^**^	0.33^**^	0.33^**^	0.20^**^	0.37^**^	1			
Social responsibility	0.24^**^	0.24^**^	0.49^**^	0.46^**^	0.56^**^	0.50^**^	1		
Impacts of technology	0.31^**^	0.21^**^	0.25^**^	0.36^**^	0.39^**^	0.19^*^	0.49^**^	1	
Knowledge of laser levelling	0.31^**^	0.26^**^	0.23^**^	0.34^**^	0.36^**^	0.20^*^	0.19^**^	0.55^**^	1

^*^significant in *p* < 0.05, ^**^ significant in *p* < 0.01

### Measurement model evaluation

As Table 7 shows, the parameters of the measurement model based on Hu and Bentler (1995) indicated that the constructs were appropriately measured.

**Table 7:** Model evaluation overall fit measurements

Goodness of fit measure	Recommended criterion	Obtained results of this research
Chi-square/degree of freedom (X^2^/df)	≤3	0.69
*p*-value	≥0.05	0.86
Normed Fit Index (NFI)	≥0.90	0.99
Non-Normed Fit Index (NNFI)	≥0.90	1.00
Comparative Fit Index (CFI)	≥0.90	1.00
Goodness-of-Fit Index (GFI)	≥0.90	0.97
Adjust Goodness-of-Fit Index (AGFI)	≥0.90	0.95
Root Mean Square Residual (RMSR)	≤0.05	0.02
Root Mean Square Error of Approximation (RMSEA)	≤0.1	0.0001

**Fig. 3:**
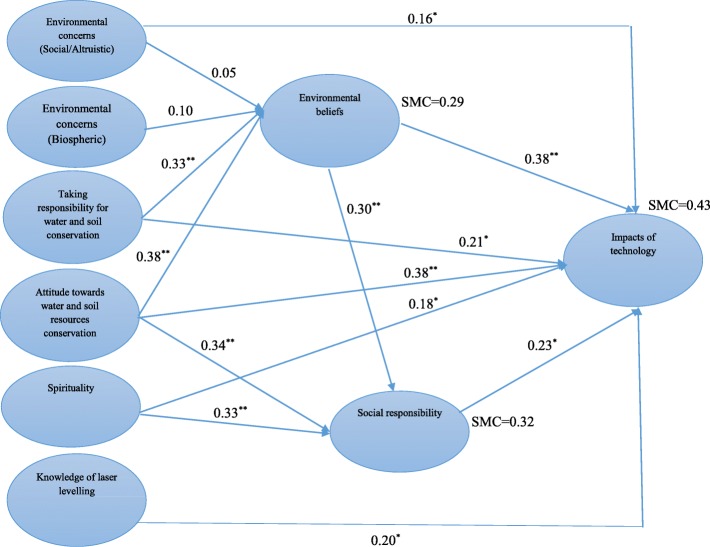
Structural equation modeling and path coefficients between variables. ^*^ significant in *p* < 0.05, ^**^ significant in *p* < 0.01

The analysis of causal effects (Fig. 3) revealed that taking responsibility regarding water and soil resources conservation had a positive effect on environmental beliefs (λ = 0.33, *p* < 0.01). The more responsibility taken towards water and soil resources conservation, the higher their environmental beliefs. Attitude towards the water and soil resources conservation (λ = 0.38, *p* < 0.01) had a positive and direct effect on environmental beliefs. Totally, these variables accounted for 29% of changes in environmental beliefs (SMC = 0.29). Based on the findings, direct effect of attitude towards water and soil resources conservation (λ = 0.34, *p* < 0.01), spirituality (λ = 0.33, *p* < 0.01), and environmental beliefs (β = 0.30, *p* < 0.01) on social responsibility were positive and significant. Furthermore, taking responsibility regarding water and soil resources conservation influenced social responsibility indirectly through environmental beliefs variable. Those variables defined 32% of changes in social responsibility (SMC =0.32). Attitude towards water and soil resources conservation and environmental beliefs had the highest direct effect on individual perception toward laser levelling impacts. The causal effect between the variables was 0.38. The causal effect of environmental beliefs is quite comparable to Stern et al. (1999).

Afterwards, social responsibility had the highest effect on individual perception (β = 0.23, *p* < 0.05). According to the findings, taking responsibility towards water and soil resources conservation had a positive and direct effect on individual perception toward laser levelling impacts (λ = 0.21, *p* < 0.05). This converges with several studies (Garling et al., 2003; Stern et al. 1999). Spirituality had a direct effect on individual perception (λ = 0.18, *p* < 0.05). Increase of knowledge of laser levelling influenced individual perception positively (λ = 0.20, *p* < 0.05). The result is consistent with Kalantari and Abdollahzadeh (2008). Environmental concerns (social/altruistic items) affected individual perception with the coefficient of 0.16 (*p* < 0.05). This result converges with that of Ibtissem (2010). The external and above-mentioned variables predict 43% of individual perception of the impacts (SMC = 0.43).

## Conclusion

Economic and social development will be achieved by policies, programs and development plans. Laser levelling technology has been implemented recently in Iran and a large amount of state budget has been allocated for it so far. According to the results, we can take into account this advanced technology in terms of environmental, economic and social sustainability. But the mean of technical and economic impacts was higher than environmental and social impacts. In such a way that mean of most economic and technological impacts is higher than 2 and this is less than 2 for environmental and social impacts. This is due to experts’ heedlessness and unawareness in relation to these impacts. Then, laser levelling may have considerable impacts on rural communities. We should note that development plans are carried out with the purpose of progress and they can be very beneficial, so their destructive and social undesirable impacts should be taken into consideration. If any strategy is not considered for direct and indirect negative impacts of the technology, it will result in undesirable consequences. One of the impacts of this technology is reducing the number of labor force, especially for irrigation operation. In the case that, authorities do not pay attention to it, many social problems will occur. When there is no job opportunity for them in other sectors, they will face many social problems during the long-term.

Another important impacts of laser levelling are decreasing irrigation period and times and consequently, decrease of water consumption. Hence, this technology can be introduced as a strategy for drought management and water shortage crisis. Moreover, the technology may result in managing of crop residues, decreasing tillage operation, and reducing machineries traverse (improving field trafficability) by using conservation tillage and zero tillage planting.

Attitude towards water and soil resources conservation and environmental beliefs had the most considerable direct effect on perception of the impacts. As well, social responsibility, taking responsibility for water and soil resources conservation, spirituality, individual knowledge toward laser levelling, and environmental concerns (social/altruistic) affected the impacts. The Value-Belief-Norm (VBN) model proposes that environmental beliefs influence awareness of consequence. Different people have concerns about environmental issues and have shown pro-environmental behavior because they believe in and are concerned about adverse consequences of environmental problems for themselves, others, or the biosphere. Therefore, encouraging the psychological variables related to personality features of individuals and their motivations in order to modify individual perception is necessary.

Due to the effect of individual knowledge on individual perception toward impacts, raising knowledge is required. In order to increase the knowledge, planning in-service courses for experts, organizing a network of specialists, educators and experts, developing training programs for experts, creating learning groups and providing conditions for group discussion to facilitate learning of laser land levelling are suggested. Having a positive attitude toward water and soil resources conservation is considered as a factor leading to a higher perception of laser levelling impacts. Empowering experts via developing training programs is required for changing the attitude towards water and soil resources conservation so that it should be considered by the authorities.

## Data Availability

The datasets generated and/or analyzed during the current study are not publicly available due [Because all of the data was gathered by the research team] but are available from the corresponding author on reasonable request.

## Abbreviation

AGFI: Adjust Goodness-of-Fit Index
CFI: Comparative Fit Index
GFI: Goodness-of-Fit Index
NFI: Normed Fit Index
NNFI: Non-Normed Fit Index
RMSEA: Root Mean Square Error of Approximation
RMSR: Root Mean Square Residual
VBN: Value-Belief-Norm
